# Influence of Thermal Treatment and Granulometry on Physicochemical, Techno-Functional and Nutritional Properties of Lentil Flours

**DOI:** 10.3390/foods13172744

**Published:** 2024-08-29

**Authors:** Angela Daniela Carboni, Gonçalo Nuno Martins, Paula Cristina Castilho, María Cecilia Puppo, Cristina Ferrero

**Affiliations:** 1 CIDCA—Centro de Investigación y Desarrollo en Criotecnología de Alimentos, Facultad de Ciencias Exactas, Universidad Nacional de La Plata (UNLP–CONICET), Calle 47 y 116, La Plata 1900, Argentina; 2 CQM—Centro de Química da Madeira, Universidade da Madeira, Campus da Penteada, 9020-105 Funchal, Portugal

**Keywords:** lentils, granulometry, hydration properties, dietary fiber, thermal treatments

## Abstract

Legume flours are an increasingly popular food ingredient. Thermal treatments applied prior to milling legumes and granulometry can modify flour properties, altering sensory, digestibility and functional attributes. Raw and treated (soaked and cooked) lentil flours of different granulometry were produced. The applied treatment resulted in an increase in fiber content (25.4 vs. 27.6% for raw and treated lentil flour, respectively) and water absorption capacity. It also led to a decrease in ash content (3.3 vs. 1.8% for raw and treated, respectively) and a darker flour. Treated lentil flour was mainly composed of fractions of high granulometry, which could be beneficial for products where a lower glycemic index is sought, as they demonstrated higher fiber and lower carbohydrate content than the finer fractions. Treated flour may be used as an ingredient in the development of raw products, including beverages and desserts, due to its reduced anti-nutritional compounds’ content and enhanced organoleptic aspects. The obtained results allow an in-depth characterization of raw and treated lentils flour with different particle sizes to consider a formal and complete standardization of these flours and for understanding their utility and specific food applications.

## 1. Introduction

Legumes and their flours are being increasingly used in the development of food products, such as baked goods, meat products, yogurts, vegetable drinks, and desserts, among others [1,2,3]. Despite the usefulness of incorporating legume flours in the development of nutritious foods, certain aspects must be taken into account. Legume seeds can undergo different pre-treatments (soaking, cooking, germination, or extrusion) before grinding, which can lead to improvements in the nutritional, technological and sensory attributes of the flours per se or the products made with them [4,5,6]. Thermal treatments applied to legumes reduce their called “anti-nutritional” components, change protein and starch digestibility, as well as fiber content, and some functional properties [7,8]. Moreover, these procedures can enhance sensory characteristics of legumes by reducing their undesirable flavors [9,10].

Lentils are a cheap and sustainable protein and fiber source, low in fat and sodium [11,12]. They are a gluten-free ingredient and their consumption can help in the prevention of some health conditions [13,14]. Furthermore, lentil flours can offer interesting technological properties for food development [15]. Anti-nutritional compounds of lentils include trypsin inhibitors, tannins and phytic acid, among others [6]. These factors can have a negative impact on consumer’s health, reducing protein and mineral availability, causing undesirable gastrointestinal symptoms and, in some cases, affecting the growth of some organs [16,17]. Although lentil flour is increasingly being used in the production of baked goods [18,19], detailed information on the production conditions of this ingredient remains scarce, while various quality aspects of other flours are well described. For example, wheat flour, which is one of the most widely used flours in the world, is well standardized as regards its functionality and composition.

The particle size of a flour can affect several characteristics of food products [20,21]. In general, coarser flour fractions tend to inhibit the attack of digestive enzymes, while flours with finer granulometry are more prone to cell rupture and therefore offer greater nutrient bioavailability [22]. When evaluating the in vitro carbohydrate digestion of legume flours with different particle sizes, Kathirvel et al. [23] found that coarser granulometry resulted in a reduction in glucose release, which can be beneficial for several health conditions. Furthermore, particle size can change the capacity of a type of flour to absorb and retain certain components, like water or oils. Studies on legume flours with various particle sizes include pulses such as chickpeas, beans or peas [24,25,26,27,28]. Regarding lentils, the effect of different particle sizes on the characteristics of red lentils’ flour have been more extensively studied [22,29,30,31], than on brown lentils’ flours (laird lentil) [23]. Red and brown lentils have major differences, including shorter cooking times for the red variety and different flavors. However, there appears to be no formal categorization dividing lentil (and other legumes) flour fractions into fine and coarse. Bourré et al. [30] highlighted the importance of standardizing and disclosing legume flours’ quality aspects, including particle size, to achieve a certain level of consistency for consumers. Furthermore, knowing the functionality of flours with different particle sizes makes it possible to use them in food production for different purposes. To this end, more information on lentil flour, including its functional and technological properties, as well as a complete nutritional characterization can be useful to make it an emerging ingredient of choice for food development. Additionally, knowing the influence of possible pre-treatments applied to lentils, would be beneficial to obtain optimal food products [31].

The objective of this study was the evaluation of the physicochemical, techno-functional, and nutritional characteristics of heat-treated lentil flour compared to raw lentil flour produced from brown lentils, as well as to assess the influence of granulometry in the named characteristics.

## 2. Materials and Methods

### 2.1. Materials

Raw lentils (*Lens culinaris* Medikus var. variabilis) were provided by Don Elio (Santa Fe, Argentina). The composition provided by the manufacturer was as follows: 22 g protein/100 g, 12 g fiber/100 g, 54 g carbohydrates/100 g, and 15 mg sodium/100 g. Lipids were not detected. The seeds were kept at 25 °C under low humidity conditions throughout the making of this work.

### 2.2. Methods

#### 2.2.1. Obtention of Lentil Flour

Treated lentil flour (TLF) was produced according to Carboni et al. [19]. Raw lentils were soaked (8 h–25 °C) in distilled water in a 1:5 lentil-to-water ratio and were then boiled (30 min–100 °C) using the same amount of freshly added water (1:5 lentil-to-water ratio). After these procedures, the lentils obtained were dried (12 h–60 °C) and ground in a coffee grinder (Peabody, Hurlingham, BA, Argentina) in 3 intervals of 15 s each. This treatment was selected considering the time necessary to reduce the flour particles to a size suitable for its incorporation in baked goods. To evaluate the effect of soaking and cooking treatments on the flour’s properties, raw lentil flour (RLF) was produced by grinding raw lentils.

#### 2.2.2. Particle Size Distribution of Flour

TLF was fractionated with a set of sieves obtaining five fractions of different granulometry. For the fractions’ separation, 25 g of TLF was introduced in the first sieve and shaken vigorously. Fractions 1 to 5 (F1–F5) varied in particle size (mm) as follows: F1 ≥ 1.0; 1.0 > F2 ≥ 0.50; 0.50 > F3 ≥ 0.250; 0.250 > F4 ≥ 0.125; and 0.125 > F5.

#### 2.2.3. Color of Lentil Flours

The color of TLF, of its corresponding fractions, and of RLF was evaluated with a colorimeter (Chroma Meter CR-300C, Minolta, Tokyo, TK, Japan) by the placement of flour samples on glass plates, taking care to cover the entire surface of the plate in order to avoid measuring errors. The Hunter parameters L*, a*, and b* are obtained: L* is associated with luminosity (with values going from 0 to 100 representing black to white), while a* indicates redness (positive values) to greenness (negative values), and b* is correlated to yellowness (positive values) and blueness (negative values). Color was evaluated five times for each sample. The Browning index (BI), a measure associated with the intensity of the brown color, was calculated from the obtained data according to Equation (1) [32]:

(1) $$BI=\frac{100\times \left(x-0.31\right)}{0.172}$$

where *x* is calculated according to Equation (2) [32], after determining the L*, a*, and b* values.

(2) $$x=\frac{a^{*}+\left(1.75\times L^{*}\right)}{\left(5.465\times L^{*}\right)+a^{*}-\left(3.012\times b^{*}\right)}$$

#### 2.2.4. Hydration and Absorptive Properties of Flours

Moisture of flours was evaluated in triplicate using a moisture analyzer (DBS 60-3, Kern & Sohn GmbH, Balingen, BW, Germany). Water activity (*a_w_*) of each flour was evaluated at 25 °C in triplicate using an Aqualab 4TEV meter (Decagon Devices Inc., Pullman, WA, USA). Water holding capacity (WHC) was determined as follows: one g of each flour was placed in pre-weighed tubes, to which 10 mL of distilled water was added. The tubes were left to rest (30 min–25 °C) and vortexed every 5 min to ensure the complete hydration of the samples. Tubes were centrifuged (5000× *g*–10 min) and unabsorbed water (i.e., the supernatant) was discarded. WHC was calculated as the g of water absorbed per g of flour [33]. The oil holding capacity (OHC) refers to the ability of a sample to absorb oils. The procedure for OHC determination was identical to WHC determination but using 10 g of sunflower oil (Continente, Lisboa, LIS, Portugal) rather than water.

#### 2.2.5. FTIR of Flours and Carbohydrate Analysis

Differences in the carbohydrate composition of RLF, and TLF and its fractions were assessed by Fourier transform infrared (FTIR) spectrometry. The FTIR spectra of the flours were collected using Spectrum Two (Perkin Elmer, Waltham, MA, USA) equipment with attenuated total reflectance (ATR) apparatus with a diamond crystal (UATR Two, Perkin Elmer, Waltham, MA, USA). Spectra were registered in the 4000–400 cm^−1^ range by co-adding 32 scans with 4 cm^−1^ spectral resolution at 20 °C. The obtained spectra were evaluated with the Spectrum software (Perkin Elmer, Waltham, MA, USA). Carbohydrates were then extracted from lentil flours following the methods described by Fruehwrith et al. [34] and Prieto-Santiago et al. [35] with modifications: 100 mg of flour was extracted using 5 mL of distilled water at 80 °C and incubated (60 °C–30 min–stirring at 200 rpm). After extracting, the supernatants were removed after 10 min of centrifugation at 5000 rpm, filtered using 0.45 µm cellulose acetate syringe filters, and analyzed by High-Performance Liquid Chromatography with Refractive Index detection (HPLC-RI) as described according to Martins et al. [36]. The °Brix (total dissolved solids) value of the extracts was determined using a RX-1000 refractometer (Atago, Mumba, MH, India). The carbohydrate-containing supernatants were lyophilized and analyzed by FTIR-ATR as conducted with the flours.

#### 2.2.6. Microscopy Analysis of Flours

To evaluate the components of the different lentil flour samples, they were subjected to a Confocal Laser Scanning Microscopy (CLSM) analysis. Samples were stained with a solution of different fluorophores: Rhodamine B (0.01 g/L) for the observation of proteins, calcofluor white (0.1 g/L) for fiber components, and fluorescein isothiocyanate (FITC) (0.1 g/L) for starch granules. A portion of flour with the fluorophore solution was placed in a glass slide and it was put to rest (1 h–dark conditions). Samples were rinsed and observed in a confocal microscope Olympus FV-1000 (Olympus, Tokyo, TK, Japan). The microstructure of the different lentil flour fractions was also studied by Scanning Electron Microscopy (SEM) using a Phenom ProX equipment (Phenom World, Eindhoven, NB, The Netherlands) at 15 kV.

#### 2.2.7. Thermal Analysis

A Q100 differential scanning calorimeter (DSC) (TA Instruments-Waters LLC, New Castle, DE, USA) was used to evaluate the thermal transitions that occur in RLF and TLF when exposed to heat. Briefly, 20% flour aqueous suspensions were prepared one day before the tests and allowed to rest (24 h–4 °C). Moreover, 6 mg of each dispersion were weighed in capsules and hermetically sealed. An empty capsule was used as a reference. Samples were heated from 10 to 130 °C with a heating ramp of 10 °C/min. The enthalpy of the thermal transition was obtained.

#### 2.2.8. Nutritional Profile of Flours

Total protein, lipid, dietary fiber, ash, and moisture of flours were determined according to methods 46-12.01, 30-10.01, 32–05.01, 08-01.01, 44-15.02 of the AACC, respectively [37]. The factor of conversion for proteins was 6.25. Carbohydrate content (different from fiber) was calculated by difference, while energy content was estimate through Atwater coefficients. Mineral content (Ca, Fe, K, P, Na, Zn, and Mg) was evaluated with an Inductively Coupled Optical Emission Plasma Spectrometer (ICP-OES) in TLF after its digestion in an acidic medium.

### 2.3. Statistical Analysis

The obtained results were evaluated by one-way analysis of variance (ANOVA). When this analysis expressed statistical differences (*p* < 0.05), intragroup comparisons were tested by the Tukey test.

## 3. Results and Discussion

### 3.1. Particle Size Distribution of Lentil Flour

**Figure 1:**
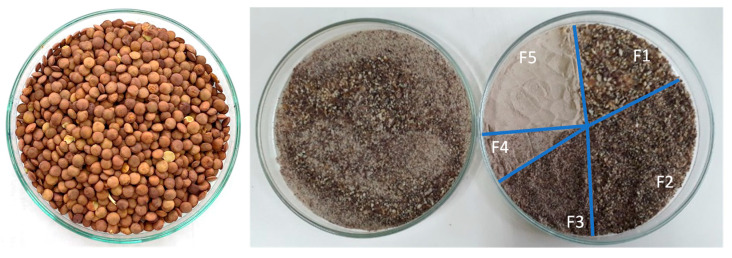
Raw lentils (**left**), TLF (**middle**) and distribution of its fractions (**right**). F1 = coarsest fraction with particle size ≥ 1 mm; F2 = fraction with particle size: 1 mm > size ≥ 0.5 mm; F3 = fraction with particle size: 0.5 mm > size ≥ 0.250 mm; F4 = fraction with particle size: 0.250 > size ≥ 0.125; F5 = finest fraction with particle size < 0.125.

Figure 1 shows the aspect of the raw lentils used for lentil flour obtention the TLF (obtained after soaking, cooking and grounding) and its different flour fractions according to their distribution percentage detailed in Table 1. TLF is very heterogeneous in terms of particle size and appearance because the constituent fractions (F1 to F5) are substantially different in these aspects. F1 represents the coarsest fraction and shows a heterogeneous aspect with particles of various shapes. Contrarily, F5 (finer fraction) presents homogenous features. The predominant fractions were F2 and F5, each representing more than 25% of the flour’s composition, while the least present fraction was F4. The use of flours with different particle sizes impacts the final quality of products. Marchini et al. [22] evaluated lentil flour fractions, finding that those with particle size of ~200 µm led to better results during bread making, when comparing with samples with finer fractions. de la Hera et al. [20] proposes that lentil flour with fine particle sizes (<140 µm) can enhance cake attributes, including an augmentation in specific volume, a reduction in cake hardness and an increment in cohesiveness. In this sense, based on the desired characteristics of the specific final product of interest, the grinding of legumes or other ingredients should be adjusted to obtain the ideal particle-sized ingredient. However, classifying flours as having fine or coarse particle sizes is not easy because these definitions vary between authors.

### 3.2. Color of Lentil Flours

Table 2 shows the obtained color parameters’ values of TLF, its fractions, and RLF. Each sample’s Hunter parameters (L*, a*, and b*) were translated using Nix Color Sensor Software(Nix Sensor Ltd., Hamilton, ON, Canada) into an RGB code to produce the colors shown, which resemble the real-life colors captured in Figure 1. F1 shows a heterogeneous aspect with different color tones. Contrarily, F5 presents homogeneous light-brownish particles. RLF, TLF, F4, and F5 showed values of L* (indicator of luminosity) > 50, while F1, F2, and F3 presented the results of L* < 50, which translates in a darker shade for these fractions’ color. Ahmed et al. [23] described an augmentation in L* in two varieties of lentil flour when particle size became finer. The a* parameter is positive in all samples, indicating their color has a greater redness than greenness character, but their low values indicate their small contribution to the color of the flours. The b* parameter being positive showcases the yellowness of the color and explains the difference in color for the RLF. Similar results were obtained by Bragança et al. [38]. TLF’s color most resembled that of F4, curiously the fraction least present in terms of particle size distribution (<10%). This could be probably related to the heterogeneity of TLF, as seen in Figure 1, which can interfere in the precision of color measurement. Additionally, F4 is an intermediate fraction, so TLF can present an intermediate color between the other fractions, and F5 (representing one of the majority fractions) could partially compensate for the darkness of the coarser fractions (F1 to F3).

The BI results tended to be lower in TLF, F4, and F5 than in the rest of the samples, probably because of the differential distribution among fractions of the dark-colored tegument. A higher degree of milling probably leads to the loss of lentil pigments [31]. In general, it can be said that no clear trend in particle size on the color of the samples was observed. Depending on the matrix in which it will be used and whether or not it will be combined with other flours, the color of each flour will have a different effect on the product’s final quality. Therefore, if the presence of lentil flour is to be masked, it would be advisable to use the finer fractions, the color of which could be more similar to traditional flours such as wheat flour.

### 3.3. Hydration and Absorptive Properties of Lentil Flours

Table 3 presents the results of the moisture content, *a_w_*, WHC, and OHC of the different lentil flour samples. The water holding capacity (WHC) represents the capacity of a sample to hold water against centrifugation, gravity or during the application of processes like heating [39]. It can affect the texture of foods to a great extent [40]. Drying treatment applied to lentils modified the moisture of the obtained lentil flour. RLF showed the highest moisture, while F1 presented the lowest. Something similar occurred regarding *a_w_* for RLF. This parameter is related to the water availability for enzymatic reactions, microbial growth, spore germination and the production of toxins [41]. Previous work [36] analyzed the carbohydrate content of the soaking and cooking waters of lentil seeds. Up to 1.73% of carbohydrates were removed from the plant material, lowering the number of hydrophilic compounds present in the seeds. Lower *a_w_* values can be favorable for preserving food products, and all samples showed good *a_w_* results since undesirable reactions commonly occur at *a_w_* > 0.6 [42].

The OHC did not change among samples, while the WHC was higher for treated flours. When evaluating yellow lentil, Odabas et al. [5] found that heat treatment augmented the WHC of these samples. Nagai et al. [43] evaluated the WHC of beans, finding significantly lower values (between 1.4 and 1.9 g water/g flour) than those from the present work. According to Kerr et al. [21], finer particle-sized legume flours possess a lower water absorbing capacity. However, this parameter and its variation with particle size can change depending on the legume evaluated [30]. Despite the moisture content and *a_w_* values being lower in the treated lentils flours, the WHC was higher. This behavior may be due to starch gelatinization occurring during the thermal treatment, leading to higher WHC of the flour [44,45].

### 3.4. FTIR of Flours and Carbohydrate Analysis

**Figure 2:**
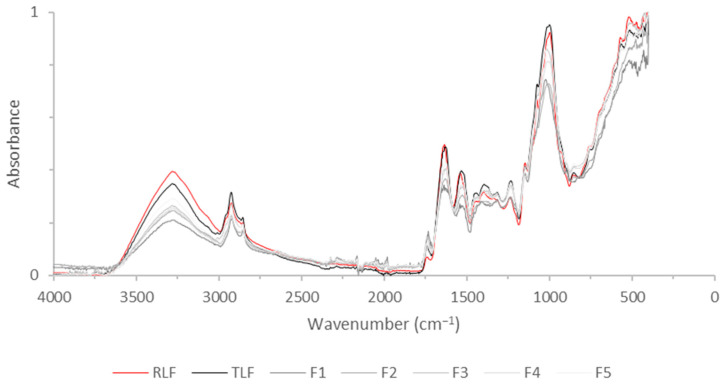
IR spectra of lentil flours. RLF = raw lentil flour; TLF = treated lentil flour; F1 = coarsest fraction with particle size ≥ 1 mm; F2 = fraction with particle size: 1 mm > size ≥ 0.5 mm; F3 = fraction with particle size: 0.5 mm > size ≥ 0.250 mm; F4 = fraction with particle size: 0.250 > size ≥ 0.125; F5 = finest fraction with particle size < 0.125.

**Figure 3:**
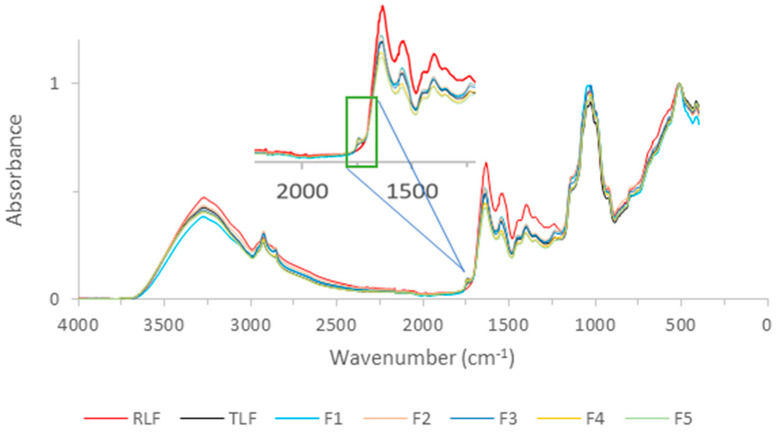
IR spectra of the lentil flours’ lyophilized extracts. RLF = raw lentil flour; TLF = treated lentil flour; F1 = coarsest fraction with particle size ≥ 1 mm; F2 = fraction with particle size: 1 mm > size ≥ 0.5 mm; F3 = fraction with particle size: 0.5 mm > size ≥ 0.250 mm; F4 = fraction with particle size: 0.250 > size ≥ 0.125; F5 = finest fraction with particle size < 0.125.

Figure 2 shows the IR spectra of the thermally treated and untreated lentil flours, while the IR spectra of the lyophilized supernatants of the flour extraction are shown in Figure 3. The detected bands in Figure 2 are mainly due to polysaccharides and other carbohydrates (at 1000 cm^−1^), which make up the cell wall of the plant [46]. Lipids (3000–2800 cm^−1^ corresponding to the C-H bending of long-chain fatty acids) and protein-related bands (corresponding to Amide A at 3300 cm^−1^, Amide I at 1640 cm^−1^, and Amide II at 1540 cm^−1^) were also detected [46,47]. 

The spectra for all samples are very similar regarding the detected bands because the thermal treatments did not cause any fundamental changes in the composition of the material. However, differences are observed in the spectral intensity of the bands, especially at 3300 cm^−1^ and 1000 cm^−1^, differences that are more noticeable between the RLF and some of the treated samples (such as F1). Chávez-Murillo et al. [46] evaluated the effects of a thermal treatment employing water on different legumes, finding changes in the spectra intensity between raw and cooked samples. Treated and untreated chickpea flour were studied by Kotsiu et al. [47]. These authors suggest that changes in the intensity of this carbohydrate region could be attributed to different amounts of starch present in the legume samples.

The bands detected on the legume flours and on their supernatants were practically the same, with the exception of a band observed in the treated lentils flours (TLF, F1 and F5) at 1750 cm^−1^, which seems to be higher in Figure 2 than in Figure 3. This region is related to the presence of pectin or esters [48]. Pectic substances have been extracted from the cell walls of lentils subjected to cooking and were extracted more in well-cooked samples [49]. This peak at 1750 cm^−1^ is visible in the FTIR spectra of RLF in Figure 2 but not in its extract in Figure 3, indicating that soaking and cooking were necessary to extract pectic substances and make them detectable in the extracts of the flours. The HPLC-RI analysis corroborates this notion. The individual IR bands of the evaluated flours and the lyophilized supernatants, as well as the identification of each band, are summarized in Table 4.

**Figure 4:**
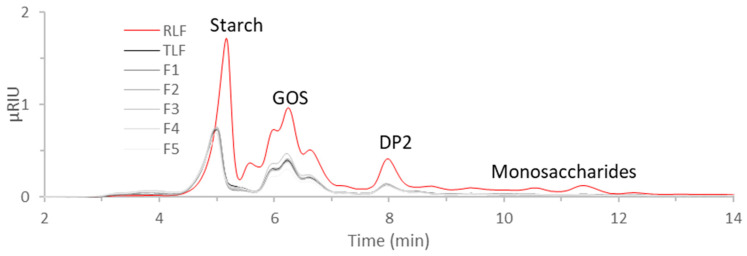
HPLC-RI chromatograms of the extracts produced from lentil flour. RLF = raw lentil flour; TLF = treated lentil flour; F1 = coarsest fraction with particle size ≥ 1 mm; F2 = fraction with particle size: 1 mm > size ≥ 0.5 mm; F3 = fraction with particle size: 0.5 mm > size ≥ 0.250 mm; F4 = fraction with particle size: 0.250 > size ≥ 0.125; F5 = finest fraction with particle size < 0.125; GOS = galacto-oligosaccharide; DP2 = degree of polymerization 2.

In the HPLC-RI chromatograms in Figure 4, various carbohydrates, including starch, galacto-oligosaccharides (GOSs), sugars with a degree of polymerization (DP) of 2 (such as disaccharides), and monosaccharides like glucose, were identified. All of these sugars were previously identified in the water discards from the soaking and cooking of lentils, finding that these sugars could be re-used as prebiotics for the growth of lactic acid bacteria [35]. Besides the usefulness of re-using these sugars, the removal of anti-nutritional compounds present in lentil flour samples through soaking and cooking treatments could be beneficial to enhance the bioavailability of nutrients (including proteins) and reduce unpleasant aromas and flavors [16,50]. The peaks found between 3 and 4 min for TLF and F1 through F5 (Figure 4), which do not appear for RLF’s extract, have been found in previous analyses of pectin samples by our group.

Also, the peak at 5 min attributed to starch can also be attributed to pectin in the TLF and its fractions’ extracts since these peaks are slightly deviated at lower retention times, and most polysaccharides produce peaks that co-elute at this retention time [51].

The higher intensities observed in the RLF’s chromatogram indicates the extraction was more effective than for TLF and its fractions. This was confirmed by the total area of the chromatograms and the °Brix values determined as an indication of the total dissolved solids, given in Table 5. The correlation between both determinations indicates the extracted content mainly consisted of carbohydrates that were detected by the HPLC-RI analysis. Other extracted components may consist of protein and lipids according to the proximal nutritional and FTIR analysis of Figure 3. The extraction procedure released more soluble components in RLF (4.6%) than in the TLF (0.7%) or its fractions. These results prove that the soaking and cooking treatments diminished the carbohydrate content of TLF, in comparison to RLF, as suggested by the nutritional compositional analysis of the flours shown in Table 6. As already mentioned, in our previous work [35], it was determined that up to 1.7% of saccharides were removed from the legume after soaking and cooking. Contrary to the results obtained by Kathirvel et al. [23], there was no difference in monosaccharides’ content, including glucose, between fractions (F1 to F5), probably because the soaking and cooking treatments had already removed these compounds that were still found in RLF’s extract and therefore in RLF itself.

### 3.5. Microstructure of Flours

**Figure 5:**
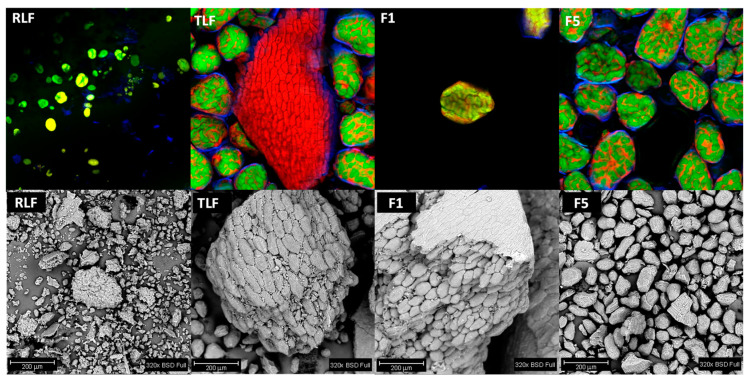
Top: Images of Confocal Scanning Laser Microscopy of lentil flour, augmentation at 20×, all fluorophores activated. Components in red represent proteins, components in green represent starch and components in blue represent fiber. Bottom: Scanning Electron Microscopy at 320×. RLF = raw lentil flour; TLF = treated lentil flour; F1 = coarsest fraction with particle size ≥ 1 mm; F2 = fraction with particle size: 1 mm > size ≥ 0.5 mm; F3 = fraction with particle size: 0.5 mm > size ≥ 0.250 mm; F4 = fraction with particle size: 0.250 > size ≥ 0.125; F5 = finest fraction with particle size < 0.125.

Images present in Figure 5 were the result of the lentil samples’ microstructure evaluation through confocal (top) and SEM (bottom) techniques. Structures in green are stained by the FITC fluorophore and correspond to starch granules. Particles in red are stained by rhodamine B and mainly correspond to the hydrophobic areas of proteins [52,53]. Particles in blue (stained by calcofluor white) correspond mainly to fiber components. The yellow color observed in some of the images, especially in F1, could be due to the superposition of parts stained by green and red. As can be seen in the confocal images, starch granules present in RLF exhibit a smaller size than in the treated flours (TLF, F1, and F5). This is due to the fact that starch granules probably suffer a gelatinization process during the treatment of lentils (Section 2.1). The granules absorb water when they are mixed, and when this suspension is heated, the gelatinization of starch occurs. Protein molecules in RLF are not so visible, while they are easier to identify in the rest of the samples. In TLF, a big proteinaceous structure surrounded by fiber can be seen, probably corresponding to a tissular fragment. In TLF and their fractions, starch granules are trapped by the proteins, while in RLF, this phenomenon is not perceivable. The higher number of particles seen in F5 compared to F1 is related to the sample itself. Due to its larger particle size, it is possible to focus the image on a single particle in F1 (Figure 1, F1). On the other hand, the flour particles are closer in F5, so a greater number of structures are observed. More than 20 particles of F5 can be counted vs. 1 particle for F1, which is coherent with the fact that F5’s particles (<0.125 mm) are at least 8× smaller than F1’s (≥1 mm). Fiber components are present in all samples, surrounding the starch granules, even in F5, which is the sample with the lowest fiber content.

SEM images showed that TLF and F1 are composed of F5 particles that detach from the main structure with grinding. Maçãs et al. [54] evaluated pea flour through SEM analysis, finding that samples with a large particle size are related to the existence of protein and starch aggregates to a greater extent than flour with fine granulometry. Ahmed et al. [31] obtained similar SEM images. RLF presents a rougher aspect compared to TLF, which shows smoother features on its particles.

### 3.6. Nutritional Profile of Lentil Flours

Considering the above results and the fact that fractions F2 to F4 did not differ significantly among themselves, it was decided to include only the following samples in the nutritional characterization: RLF, TLF and fractions F1 and F5. The amounts of each nutrient are shown in Table 6. The protein content was similar for all the analyzed samples and the results are comparable to other reports [20,55]. Hefnaway [56] evaluated samples of raw and cooked (boiled, microwaved, and autoclaved) lentils, finding no differences in protein content. However, Nosworthy et al. [8] showed that the protein content increased when lentils were cooked due to the removal of antinutrients and other solids, resulting in the protein having higher mass balances, which was not observed in the present study. Regarding lipid content, it was higher for samples that received the thermal treatment. In contrast, particle size (F1 vs. F5) had no significant effect on protein, but lipid content was affected.

TLF showed a slight increase in dietary fiber with respect to RFL. Odabas et al. [5] also found that thermal treatment applied to lentil flour led to an augmentation in fiber compared with raw flour. These authors attributed the increase in the TDF content of heat-treated lentils to resistant starch formation during cooking. F5 showed the lowest value of fiber, explaining its higher carbohydrate content (calculated by difference to the other components) and energetic value. A more thorough milling of the flour can impact dietary fiber’s amount, considering that smaller particle sizes are associated with more cell wall ruptures, which facilitate digestive enzymatic attacks [22]. Wang et al. [57] also found a decrease in the fiber content of quinoa with a higher milling degree. Carbohydrates and proteins were the major components of these samples, results that are consistent with the IR spectra of Figure 2.

Ash content was lower in TLF than in RLF, which can be related to mineral lixiviation occurring during the soaking of lentils in the production of TLF [37]. Shafi et al. [58] obtained the same trend in chestnut flour. The mineral composition of lentil flour resulted in similar to values reported by the USDA [55] for cooked lentils. The found levels of the main minerals Ca, Fe, K and Na in TLF were (in mg/100 g of flour) as follows: 77.34 ± 1.88; 9.9 ± 0.15; 169.69 ± 1.79; 16.90 ± 0.16, respectively.

Total carbohydrates different from fiber are mainly composed of starch and oligosaccharides as was revealed from the HPLC results (Figure 4). F5 possess higher starch content than the rest of the samples. DSC assays revealed that starch was partially gelatinized during the heat treatment of lentils. The enthalpy involved in the thermal transition was 4.60 J/g RLF, while the value diminished to 1.56 J/g TLF after the processing of lentils. These results showed a similar trend to those found when studying other legumes subjected to thermal treatments [47]. Nevertheless, this increase in TDF would suggest that despite partial gelatinization, the formation of a small amount of resistant starch is possible.

The present results demonstrate that the soaking and cooking treatments of lentils prior their milling led to changes in the nutritional composition of the flour, mainly in the content of fiber and ash. As described in Section 3.3**,** these treatments are also meant to decrease the anti-nutritional factors of lentil (including trypsin inhibitors, phytic acid and tannins), which can lead to better protein digestibility and higher availability of other nutrients like calcium, as well as reduction in unpleasant gastrointestinal symptoms [6,50]. On previous studies regarding the obtention of baking goods with addition of RFL, our laboratory found displeasing aroma and flavors in the final products that were not present when TLF was used [59]. Legume flours are currently available in local markets of different countries, and considering the costs of production, it is probable that in many cases, these flours are obtained from raw legumes. Currently, regulations regarding safe amounts of anti-nutritional compounds do not exist, and some of these substances are found in commercial products [48], which in certain cases can led to the hypertrophy of specific organs [17]. In this sense, TLF could be beneficial as an ingredient in products that are not subjected to cooking or other thermal treatments since this treatment has already been already performed. TFL may be applied as an additive (thickener or gelling agent) in several products, including juices, vegetable beverages, yogurts and desserts. Regarding differences in granulometry, the use of the diverse fractions or the whole treated flour contributed similar levels of protein, but the TDF, mineral and starch contribution varied. Thus, they can be used as differential ingredients according to the product requirements.

## 4. Conclusions

Lentil flour made from soaked and cooked lentil seeds was evaluated according to its techno-functional, physicochemical, and nutritional characteristics and compared with lentil flour without this thermal treatment. The granulometry of TLF was also assessed to understand the effect of particle size on the flour’s properties. TLF was mainly composed of fractions of high granulometry. A lighter flour color was seen on RLF and on finer fractions, which could be useful for products in which lentil flour is combined in a subtle way with other light-colored flours, such as wheat or rice flour. The thermal treatment led to reduced moisture but increased the capacity to absorb water while OHC did not present changes with either the treatment or particle size. The microstructure of the flour changed with soaking and cooking, leading to bigger starch granules trapped by a protein network. The thermal treatment applied to lentils increased the fiber and lipid content and reduced the ash content. TLF would be an interesting option to obtain products with a good amount of fiber and protein and that need to retain large amounts of water. This treatment could also be useful to decrease lentils’ anti-nutritional compounds and enhance protein digestibility and organoleptic aspects. TLF would be appropriate as an ingredient or additive for the development of products that do not require cooking, like beverages, yogurts and desserts. On the other hand, RLF presented a higher content of specific carbohydrates, namely free glucose. RLF could be probably considered a high-protein and high-fiber ingredient that is easy to produce and useful when soaking and cooking steps are not possible to be applied. The coarser fractions of lentil flour were higher in dietary fiber and lower in carbohydrate content, which could be useful to produce foods with a reduced degree of enzymatic digestion and when less glucose release is sought. However, the optimal particle size for lentil flour will depend on the product in which it will be applied. The obtained data help standardize the quality attributes of lentil flour, considering that there seems to be no categorization by which lentil flour (as well as other legume flour) fractions are divided into fine or coarse fractions. These findings enable those that work with legume ingredients to assess the benefits of employing pre-treatments or achieving specific particle sizes through grinding.

## Figures and Tables

**Table 1:** Particle size distribution in the fractions of the TLF lentil flour.

Fraction and Particle Size (mm)	%
F1 ≥ 1	17.76
1 > F2 ≥ 0.5	28.73
0.5 > F3 ≥ 0.250	17.81
0.250 > F4 ≥ 0.125	9.64
F5 < 0.125	26.07

F1 = coarsest fraction; F5 = finest fraction.

**Table 2:** Color of lentil flours.

Sample	Parameter				
	L*	a*	b*	BI	Color
RLF	66.78 ± 0.42 ^c^	2.33 ± 0.18 ^a^	25.08 ± 0.14 ^d^	57.63 ± 1.26 ^d^	
TLF	58.93 ± 1.61 ^b^	2.36 ± 0.14 ^a^	15.53 ± 0.10 ^b^	40.68 ± 1.28 ^b^	
F1	48.24 ± 0.93 ^a^	3.18 ± 0.38 ^c^	16.68 ± 0.27 ^c^	54.32 ± 1.16 ^c^	
F2	47.67 ± 1.33 ^a^	4.03 ± 0.16 ^d^	16.57 ± 0.43 ^c^	57.61 ± 1.48 ^d^	
F3	47.35 ± 0.95 ^a^	5.09 ± 0.12 ^e^	15.86 ± 0.16 ^b^	56.44 ± 1.04 ^cd^	
F4	58.91 ± 0.46 ^b^	3.74 ± 0.22 ^d^	14.10 ± 0.25 ^a^	39.09 ± 1.10 ^b^	
F5	68.07 ± 0.28 ^c^	2.78 ± 0.05 ^b^	15.48 ± 0.13 ^b^	35.75 ± 0.13 ^a^	
*p*-value	<0.0001	<0.0001	<0.0001	<0.0001	

Results are expressed as mean ± standard deviation. Different letters mean significant differences between samples by Tukey test (*p* < 0.05). RLF = raw lentil flour; TLF = treated lentil flour; F1 = coarsest fraction with particle size ≥ 1 mm; F2 = fraction with particle size: 1 mm > size ≥ 0.5 mm; F3 = fraction with particle size: 0.5 mm > size ≥ 0.250 mm; F4 = fraction with particle size: 0.250 > size ≥ 0.125; F5 = finest fraction with particle size < 0.125; L* = luminosity (with 0 = black; 1 = white); a* = redness (positive values) to greenness (negative values) chromaticity; b* = yellowness (positive values) to blueness (negative values); BI = Browning index. n = 5.

**Table 3:** Hydration properties and oil holding capacity of lentil flours.

Sample	Moisture (%) *	*a_w_* *	WHC (g Water/g Flour) ^†^	OHC (g Oil/g Flour) ^†^
RLF	11.78 ± 0.07 ^e^	0.596 ± 0.005 ^e^	2.28 ± 0.02 ^a^	1.78 ± 0.15 ^a^
TLF	6.78 ± 0.11 ^c^	0.315 ± 0.007 ^a^	3.17 ± 0.08 ^bc^	1.81 ± 0.00 ^a^
F1	4.60 ± 0.21 ^a^	0.388 ± 0.007 ^b^	3.25 ± 0.18 ^c^	1.91 ± 0.11 ^a^
F2	5.81 ± 0.11 ^b^	0.301 ± 0.001 ^a^	3.18 ± 0.11 ^bc^	1.85 ± 0.18 ^a^
F3	6.89 ± 0.13 ^c^	0.407 ± 0.013 ^c^	3.21 ± 0.12 ^bc^	1.71 ± 0.11 ^a^
F4	7.20 ± 0.18 ^cd^	0.461 ± 0.001 ^d^	3.09 ± 0.06 ^bc^	1.82 ± 0.02 ^a^
F5	7.61 ± 0.17 ^d^	0.394 ± 0.002 ^bc^	2.86 ± 0.02 ^b^	1.41 ± 0.16 ^a^
*p*-value	<0.0001	<0.0001	0.0002	0.1110

Results are expressed as mean ± standard deviation. Different letters mean significant differences between samples by Tukey test (*p* < 0.05). RLF = raw lentil flour; TLF = treated lentil flour; F1 = coarsest fraction with particle size ≥ 1 mm; F2 = fraction with particle size: 1 mm > size ≥ 0.5 mm; F3 = fraction with particle size: 0.5 mm > size ≥ 0.250 mm; F4 = fraction with particle size: 0.250 > size ≥ 0.125; F5 = finest fraction with particle size < 0.125. WHC = water holding capacity; OHC = oil holding capacity. * n = 3; ^†^ n = 4.

**Table 4:** Identification of the different bands of the IR bands.

Position (cm^−1^)	Identification
3300	Amide A
3000–2800	C-H bending of long-chain fatty acids
1750	Pectic substances
1640	Amide I
1540	Amide II
1000	Carbohydrates

**Table 5:** Total area of the obtained HPLC-RI chromatograms (Figure 4), and °Brix values of each flour/fraction.

Sample	Total Area	Brix
RLF	21.1 ± 2.2 ^b^	4.6 ± 0.6 ^b^
TLF	8.8 ± 0.7 ^a^	0.7 ± 0.6 ^a^
F1	7.7 ± 0.4 ^a^	0.7 ±0.6 ^a^
F2	8.0 ± 0.6 ^a^	0.7 ± 0.6 ^a^
F3	9.5 ± 0.4 ^a^	0.7 ± 0.6 ^a^
F4	9.6 ± 1.3 ^a^	1.0 ± 0.0 ^a^
F5	7.1 ± 0.9 ^a^	0.7 ± 0.6 ^a^
*p*-value	<0.0001	<0.0001

Results are expressed as mean ± standard deviation. Different letters mean significant differences between samples by Tukey test (*p* < 0.05). RLF = raw lentil flour; TLF = treated lentil flour; F1 = coarsest fraction with particle size ≥ 1 mm; F2 = fraction with particle size: 1 mm > size ≥ 0.5 mm; F3 = fraction with particle size: 0.5 mm > size ≥ 0.250 mm; F4 = fraction with particle size: 0.250 > size ≥ 0.125; F5 = finest fraction with particle size < 0.125; n = 3.

**Table 6:** Nutritional composition of lentil flours (% d.b).

Sample	Protein *	Lipids *	TDF *	Ash ^†^	Carb •	Energy Content (Kcal/100 g)
RLF	23.50 ± 1.21 ^a^	0.87 ± 0.05 ^a^	25.42 ± 0.21 ^b^	3.34 ± 0.01 ^c^	46.87	340
TLF	23.84 ± 0.21 ^a^	1.64 ± 0.07 ^c^	27.56 ± 0.08 ^c^	1.77 ± 0.06 ^b^	45.19	346
F1	21.85 ± 0.07 ^a^	1.28 ± 0.01 ^b^	29.53 ± 0.06 ^d^	1.75 ± 0.01 ^b^	45.59	340
F5	21.20 ± 0.01 ^a^	1.55 ± 0.03 ^c^	18.99 ± 0.23 ^a^	1.52 ± 0.04 ^a^	60.59	371
*p*-value	0.1042	0.0002	<0.0001	<0.0001		

Results are expressed as mean ± standard deviation. Different letters mean significant differences between samples by Tukey test (*p* < 0.05). d.b = dry basis; RLF = raw lentil flour; TLF = treated lentil flour; F1 = coarsest fraction with particle size ≥ 1 mm; F5 = finest fraction with particle size < 0.125; TDF = total dietary fiber. Carb = carbohydrates; * n = 2; ^†^ n = 3; • = calculated by difference.

## Data Availability

The data that support the findings of this study are available from the corresponding author upon reasonable request.
